# Functional roles of magnetic nanoparticles for the identification of metastatic lymph nodes in cancer patients

**DOI:** 10.1186/s12951-023-02100-0

**Published:** 2023-09-21

**Authors:** Yuanliang Yan, Yuanhong Liu, Tongfei Li, Qiuju Liang, Abhimanyu Thakur, Kui Zhang, Wei Liu, Zhijie Xu, Yuzhen Xu

**Affiliations:** 1 Department of Pharmacy, Xiangya Hospital, Central South University, 410008 Changsha, Hunan China; 2 Hubei Key Laboratory of Embryonic Stem Cell Research, School of Basic Medical Sciences, Hubei University of Medicine, 442000 Shiyan, Hubei China; 3 Pritzker School of Molecular Engineering, Ben May Department for Cancer Research, University of Chicago, 60637 Chicago, IL USA; 4 Department of Pathology, Xiangya Hospital, Central South University, 410008 Changsha, Hunan China; 5 National Clinical Research Center for Geriatric Disorders, Xiangya Hospital, Central South University, 410008 Changsha, Hunan China; 6 Department of Rehabilitation, The Second Affiliated Hospital of Shandong First Medical University, 271000 Taian, Shandong China

**Keywords:** Magnetic nanoparticles, Metastatic lymph nodes, Iron oxide, Bioimaging, Cancer diagnosis and treatment

## Abstract

Staging lymph nodes (LN) is crucial in diagnosing and treating cancer metastasis. Biotechnologies for the specific localization of metastatic lymph nodes (MLNs) have attracted significant attention to efficiently define tumor metastases. Bioimaging modalities, particularly magnetic nanoparticles (MNPs) such as iron oxide nanoparticles, have emerged as promising tools in cancer bioimaging, with great potential for use in the preoperative and intraoperative tracking of MLNs. As radiation-free magnetic resonance imaging (MRI) probes, MNPs can serve as alternative MRI contrast agents, offering improved accuracy and biological safety for nodal staging in cancer patients. Although MNPs’ application is still in its initial stages, exploring their underlying mechanisms can enhance the sensitivity and multifunctionality of lymph node mapping. This review focuses on the feasibility and current application status of MNPs for imaging metastatic nodules in preclinical and clinical development. Furthermore, exploring novel and promising MNP-based strategies with controllable characteristics could lead to a more precise treatment of metastatic cancer patients.

## Introduction

Cancer cells could escape the primary site through complex biological mechanisms and localize to other body parts, forming metastatic tumors [1]. Carcinoma metastasis is responsible for cancer development, prognosis, and therapeutic response [2–4]. Furthermore, metastasis represents one of the leading causes of cancer-related morbidity and mortality [5, 6]. Therefore, exploring the underlying biochemical events involved in regulating the metastatic process would be essential and meaningful to identify potential targets for the inhibition and prevention of metastasis. In particular, the definition of metastatic events in the early stages can influence the clinical management of cancer patients.

It is well known that lymph node metastasis (LNM) is an important dissemination event during metastasis. Among these, the sentinel lymph nodes (SLN), the closest lymph nodes (LN) that drain the tumor area, can serve as a host of metastatic cancer cells [7]. SLN mapping has recently been used to detect early lymphatic metastases in cancer patients [8]. Traditionally, the main assessment methods for LNM are several radiomic-based technologies, including magnetic resonance imaging (MRI), computed tomography (CT), and positron emission tomography/computed tomography (PET/CT). However, these detection approaches have some significant disadvantages and limitations. MRI cannot accurately distinguish fibrosis and tumor infiltration, ultimately triggering false positive results for cancer diagnosis and stage [9]. CT scans may lead to the potential harm of exposure to ionizing radiation and the risk of overdiagnosis in patients with an incidental finding [10]. The challenges for the use of PET/CT have been raised about not only the costs and time consumption but also the misinterpretation in some noncancerous tissues with high radiotracer uptake [11].

It should be noted that although clinical imaging can efficiently determine tumor metastasis, these imaging-based techniques are challenging to detect micro-metastases, which occur early in the progression of malignancies [12]. When the findings of the imaging modalities reveal the presence of suspicious metastatic nodules, pathological tests are necessary to clarify their origin and location. An SLN biopsy should be performed for patients without nodules to confirm whether cancer cells have spread to the nearby LNs (also called regional lymph nodes, RLNs) or other organs [13]. However, pathological examination is unavoidably associated with surgical procedures that could carry the risk of harm to patients. Specifically, even after a sentinel node biopsy, some patients have been reported to have lymphedema, a pathological condition characterized by a localized accumulation of excessive interstitial fluids [14, 15]. Therefore, exploring novel noninvasive and effective strategies to better detect LNM risk in cancer patients is always of great concern.

Magnetic nanoparticles (MNPs) have been utilized to visualize the lymphatic spread of cancer cells, which is of great significance for cancer staging and treatment. The use of MNPs for human disease diagnosis was first introduced in the 1990s, serving as an agent for liver imaging, and revealed good biological safety [16, 17]. After that, since MNPs can be detected and manipulated by remote magnetic fields, their clinical application for LNM will substantially impact cancer bioimaging and early diagnosis [18, 19]. To date, iron oxide nanoparticle (IONP)-based formulations, one of the most advanced MNPs, have been used mainly as MRI contrast agents (CAs) for LN mapping [20, 21]. FDA-approved ferumoxytol, a carboxymethyl dextran-based IONP, helps detect locoregional LN in prostate cancer patients [22]. With the help of 1.5 Tesla MRI scanners, intravenous injection of ferrimagnetic or ferromagnetic IONP-labeled macrophages can specifically target LN and its metastases in a nude mouse model (BALB/c) [23, 24]. Zhou et al. constructed a dye-conjugated near-infrared (NIR) IONP, IONP-NIR830, and found that IONP-NIR830 administration could display strong T2*-weighted magnetic resonance and NIR images for preoperative and intraoperative tracking of SLN [25]. Although the application of MNPs is still in its early stages, exploring the underlying mechanisms of MNPs has excellent potential for improving the sensitivity and multifunctionality of LNM mapping. In particular, developing novel MNP-based probes for the precise assessment of LN status will significantly influence the decision-making on operative approaches and therapeutic strategies in cancer patients.

Here, we provide an updated and comprehensive review of MNPs, especially IONPs, in molecular imaging of metastatic lymph nodes (MLNs) in preclinical (Table 1) and clinical (Table 2) development. Furthermore, detailed biological characteristics and application status of different MNPs were discussed, highlighting their essential contribution to diagnosing LNM.

**Table 1:** The pre-clinical evaluation of main magnetic nanoparticles in the molecular imaging of metastatic lymph nodes

Nanoparticles	Models	Functions	Refs
Mannan-coated SPIONs	Mouse 4T1 breast tumor model	Mannan–SPION are preferentially taken up by antigen-presenting cells in metastatic LNs	[29]
SPION-antitenascin-C	Cervical cancer tissues	Specific capturing the MLNs with high tenascin-C levels	[47]
SPIOs@A-T	Mouse 4T1 breast tumor model	High imaging intensity for specific and precise detection of MLNs; No detectable biological toxicity	[48]
USPIO	Rabbit VX2 pyriform sinus carcinoma model	No reduced T2*-signal intensity for the metastatic nodules	[57]
USPIO	Dog oral cancer	No negative adverse-effects	[67]
CS015	Rabbit VX2 tumor model	Be swallowed by macrophages residing in LNs at a very small dosage	[78]
USPIO-PEG-sLe^X^	Mouse model of metastatic nasopharyngeal carcinoma	Successfully targeting E-selectin, the important factor for cancer metastasis	[82]
SPION-ICG	Swine gallbladder cancer model	The SPION-ICG dual tracer is feasible and sensitive for the SLNs detection	[89]

**Table 2:** The clinical trials of main magnetic nanoparticles in the molecular imaging of metastatic lymph nodes

Nanoparticles	Injection	Cancers	Cases	Functions	Refs
Sienna+	Peritumoral	Colorectal	27	Ex vivo confirming the nodal staging quantitatively and accurately	[13]
SPION	Subcutaneous	Breast	51	SPION is found predominantly surrounding the metastases, but not in metastatic areas	[40]
SPION	Peritumoral	Penile	2	Providing a radiation-free technique for SLN identification	[41]
SPION	Peritumoral	Esophageal	22	Determining the need for three-field lymphadenectomy with neck dissection	[42]
SPION	Intradermal	Breast	102	Diagnosis of metastatic sentinel nodes localized by CT scanning	[43]
SPION	Intradermal	Breast	150	A good correlation between SPION-MRI imaging and pathologic size	[44]
Sienna+		Penile	10	Good concordance between Sienna + and radioisotope-guided SLN mapping	[49]
USPIO		Rectal	25	Displaying higher diagnostic specificity when compared with pathologically examination	[53]
USPIO	Intravenous	Bladder/prostate	75	Could guide the surgical removal of suspicious LNs	[58]
USPIO	Intravenous	Rectal/prostate	6	Evidently improving the detection rates of LNMs smaller than 5 mm	[61]
USPIO	Intravenous	Oesophageal	9	Accurately identifying the distant and regional LNMs	[65]
USPIO	Intravenous	Oesophageal	10	More feasible and superior for LNM visualization than routine breath-hold MRI	[66]
Ferumoxtran-10	Intravenous	Gastric	17	Indicating a sensitivity of 100% with a specificity of 92.6% for nodal staging	[72]
Ferumoxtran-10	Intravenous	Gastric	31	Demonstrating the higher positive predictive value and accuracy for LNMs	[73]
USPIO-DW-MRI	Intravenous	Bladder/prostate	75	Rapidly detecting the small LNMs preoperatively	[80]

## Superparamagnetic iron oxide nanoparticles

The first IONPs, which have a size of 40–150 nm, are termed standard superparamagnetic iron oxide nanoparticles (SPIONs) [26]. The superparamagnetic phenomenon was first described in the 1950s by Néel [27], who observed the magnetic properties of small ferromagnetic or ferrimagnetic particles at the nanoscale in the external magnetic field. Typically, SPION has a core of iron oxide (Fe_3_O_4_ or Fe_2_O_3_) coated with optional aggregation preventing monomer or polymer to enhance stability. These coated materials can also be functionalized with targeting molecules or ligands to target the corresponding cells or tissues [28]. Vu-Quang et al. investigated the characteristics of mannan-coated SPIONs, which could be targeted to deliver immune cells by receptor-mediated endocytosis. They discovered that normal macrophages and dendritic cells could preferentially phagocytose mannan-SPION very early after intravenous injection [29].

**Fig. 1:**
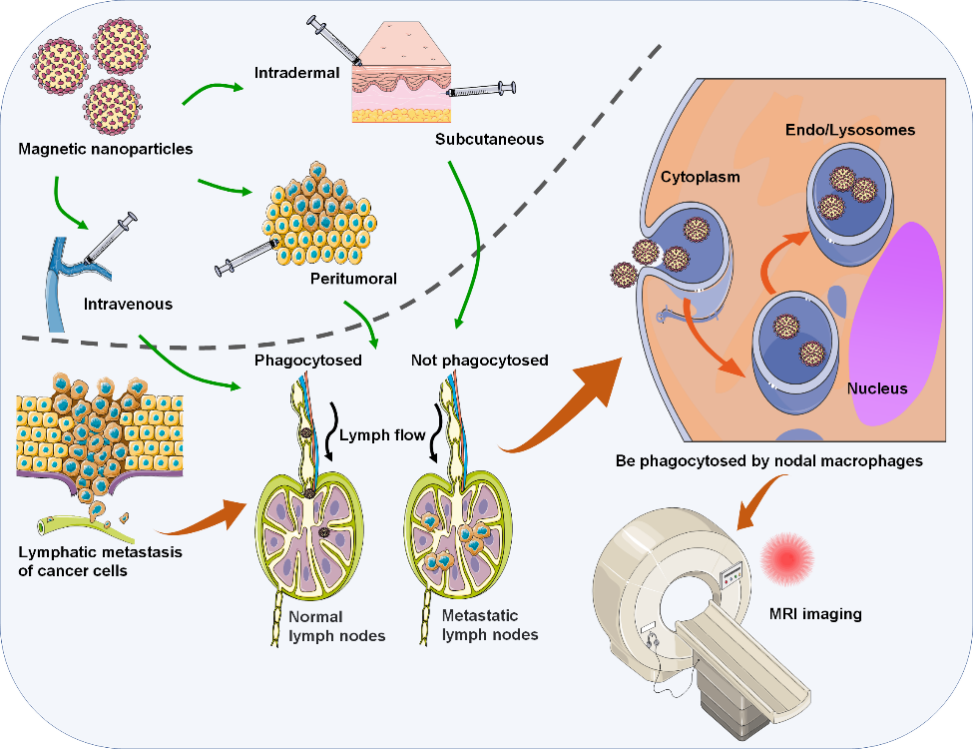
The application of MNPs-based strategies for MRI imaging of tumor lymphatic metastasis. The successful development of MNPs-based strategies in bioimaging recently made it possible to utilize MNPs as the MRI probes for MLNs tracking in cancer patients. After injection, MNPs could be preferentially phagocytosed by cells in normal lymphoid tissues, resulting in the darkening signal on T2*-weighted images. Inversely, high signal intensity on T2*-weighted images is observed for the metastatic LNs which do not take up the nanoparticles

In subsequent studies, the unique properties, such as magnetic responsiveness and high relaxivity, make SPIONs useful for their function as attractive MRI CAs in biomedical applications [30]. SPION-based MRI offers promising programs for early MLN detection while avoiding the shortcomings of radioactive labels in conventional imaging techniques [31–33]. The combination of SPION-MRI and handheld magnetometers provides a non-radioactive strategy for the preoperative [34] and intraoperative [35] locations of SLN. Preoperative injection of SPIONs in breast cancer improves detection rate and nodal yields [36]. The reliability of SLN detection by SPION-MRI incorporated with the SentiMag magnetometer has also been verified by Minamiya et al. in non-small cell lung cancer [37]. As shown in Fig. 1, the underlying mechanism is that IONPs could only be phagocytosed by reticuloendothelial cells in normal lymphoid tissues, resulting in the darkening of normal LNs in T2* weighted images. Inversely, due to the lack of reticuloendothelial cells, the tumor tissues or LNs invaded by tumor cells absorb the nanoparticles that show a high signal in the lymphoid tissues on MRI [38, 39]. Consequently, a recent study by Johnson et al. [40] confirmed that SPIONs are mainly distributed around metastatic nodules after subcutaneous injection, but are not seen within areas containing metastases. The systematic review and meta-analysis summarizing approximately 20 existing studies demonstrated that the dose, injection site, and nodal metastasis burden had no significant effect on the detection rate of LNM by SPION-based CAs. Despite this, injection of SPIONs 24 h before surgery could improve the detection rate [36].

To approve the potential use of SPIONs for magnetic resonance lymphoscintigraphy, Winter et al. [41] reported a study on the clinical validation of SPIONs for SLN identification in penile cancers. In this case, inguinal SLN could be reliably visualized by MRI after peritumoral injection of SPIONs. For patients with submucosal esophageal cancer, endoscopic injection of SPIONs into the peritumor tissues is a proper method to estimate whether to perform a three-field lymphadenectomy with neck LN dissection [42]. More importantly, these patients did not present significant side effects attributable to SPION injection. Based on the statements of Motomura et al. [43], SPION-enhanced MRI imaging showed usefulness in confirming metastatic sentinel nodes located by CT scanning. The accuracy, sensitivity, and specificity of SPION-guided imaging for detecting metastases in SLNs were 89.2%, 84.0%, and 90.9%, respectively. Subsequently, this group evaluated the correlation between SPION-enhanced MRI lymphography and pathologic size to detect metastatic SLNs [44]. They showed that for metastatic nodes with pathologic > 2 mm, the high signal intensity indicated by SPION-MRI imaging showed a good correlation with their pathological size. Given the feasible and safe properties, SPION-based magnetic procedures could propose entirely radiation-free methods for the preoperative and intraoperative location of SLNs in cancer patients [45]. Therefore, SPION-guided MRI methods are helpful for the visualization of LN mapping, guiding clinical diagnosis and treatment decisions.

It is worth mentioning here that the combination use of SPIONs and specifically targeted SPION conjugates (e.g., cell surface molecule-targeted tags) is available to depict macro- and early micrometastases within the lymphatic system. High levels of tenascin-C could be observed in cervical cancer tissues with node metastasis but not in non-metastatic patients and normal cervix tissues [46]. Synthesized SPION-anti-tenascin-C, a magnetic probe oriented specifically to tenascin-C, possessed the non-invasive ability to capture specific MLNs with high expression of tenascin-C in preoperative cervical cancer patients [47]. Wang et al. developed a novel SPIOs@A-T imaging probe to target breast cancer and ATP responsiveness to the tumor microenvironment. Mice injected with SPIOs@A-T presented high-intensity fluorescence molecular imaging and magnetic particle imaging with high sensitivity and specificity in the MLN group [48]. Recently, Sienna+, a dark brown aqueous suspension of SPIONs, has been utilized in conjunction with the handheld magnetometer SentiMag to non-radioactively locate SLNs. This magnetic nanoparticle tracer, Sienna+/SentiMag, could quantitatively and accurately confirm nodal staging, providing a feasible and safe alternative to SLN mapping in colorectal cancer patients [13]. The result of the consistency examination showed good concordance between the Sienna+/SentiMag probe and the standard radioisotope-guided dynamic SLN mapping [49]. In addition, the Sienna+/SentiMag technique enhances clinical outcomes and experience for cancer patients by reducing patient contact time, avoiding radiation exposure, and operating cost-effectively [50, 51].

These emerging imaging modalities have extended the applications of SPIONs in molecular oncological diagnostics. However, the questions regarding the pharmacokinetics and safety profiles of SPIONs and conjugates must be well addressed before the approval of these nanoparticles for clinical application in cancer patients.

## Ultrasmall superparamagnetic iron oxide

The smaller SPIONs (median diameter less than 50 nm) are called ultrasmall superparamagnetic iron oxides (USPIOs). The smaller particle size of USPIOs leads to their specific lymphotropic behavior [52]. After administration, USPIO particles could be phagocytosed by nodal macrophages and then directly drained to normal nodes. After localization to LNs, USPIOs generate a susceptibility artifact on gradient echo MRI sequences, resulting in loss of T2*-related signal [53, 54]. In contrast, high signal intensity is observed on T2*-weighted images in metastatic LNs lacking macrophages [55]. According to this guideline for USPIO-enhanced MRI, LN without blackening or hyperintense in surrounding tissues could be diagnosed as metastatic nodules. The LNs were considered non-metastatic if they displayed low signal intensity [56]. Consequently, a recent study by Shen et al. [57] evaluated the changes in the T2*-weighted image after USPIO enhancement. The results did not show a reduced signal intensity for metastatic nodules.

The preferential application of USPIO as MRI CAs in cancer patients will help improve the ability of MRI to detect LN and their metastases early and contribute to better preoperative staging. Compared to pathological morphological findings, using MRI + USPIO results in greater diagnostic specificity to identify LN status [53]. USPIO-based MRI could also provide clues for the surgical removal of suspicious LNs not included during dissection [58]. The maximum lymphographic effect could be observed after 24 h of USPIO administration [59, 60]. In general, the application of USPIO-enhanced MRI improves the detection rates of LNM, particularly for nodules smaller than 5 mm [61]. Similar studies revealed that, in patients with bladder or prostate cancer, USPIO-enhanced MRI allows for definite metastases in normal-shaped or smaller LNs [62–64]. For LN staging in patients with oesophageal cancer, administration with USPIO-enhanced MRI could accurately identify distant and regional nodules, such as celiac and mediastinal LN [65].

Furthermore, compared to routine breath-hold MRI, the MRI + USPIO method is more feasible and superior for LNM visualization in esophageal cancer patients [66]. A recent in vivo experiment suggested no negative adverse effects in dog oral cancer models treated with USPIO lymphotropic nanoparticles [67]. Despite their ability to diagnose LNM, USPIOs have not yet been approved for clinical application [68].

Several USPIO-based technologies, such as ferumoxtran-10, are being evaluated clinically and preclinically [26, 69]. Ferumoxtran-10, composed of USPIO, is a promising lymphotropic CA that could diagnose more positive metastatic LNs than traditional imaging techniques, such as PET/CT [70, 71]. Using ferumoxtran-10-enhanced MRI, Tatsumi et al. reported high accuracy in detecting LNM in 194 nodes from 17 gastric cancer patients, with a sensitivity of 100%, specificity of 92.6%, positive predictive value of 85.5%, negative predictive value of 100%, and overall predictive accuracy of 94.8% [72]. Tokuhara et al. demonstrated that the positive predictive value and accuracy for nodal staging using ferumoxtran-10 enhanced MRI in 519 nodes of 31 gastric cancer patients were 90.1% and 97.1%, respectively, significantly higher than conventional criteria, which have a positive predictive value and accuracy of 76.8% and 93.3% [73]. In addition to some tolerable side effects, such as allergic skin changes or back pain, no other side effects were observed in patients receiving ferumoxtran-10 [74, 75]. However, current reports are insufficient to support the functional roles of ferumoxtran-10 in all tumor types.

Instead, several novel USPIO-contained CAs, such as P904 [76] and PJY10 [77], and CS015 [78], have been well-developed to show better performance than ferumoxtran-10 in LNM MR lymphography in oncological fields. CS015, a polyacrylic acid (PAA) coated USPIO, enhances the long-term stability of USPIO in various solutions [79]. After subcutaneous and intravenous injections, the CS015 particles could be effectively swallowed by macrophages residing in LN, even at a very low working concentration [78]. Birkhäuser et al. evaluated the recognizable effects of USPIO-MRI combined with diffusion-weighted MRI (USPIO-DW-MRI) for metastatic LNs in patients with bladder and prostate cancer. The combined USPIO-DW-MRI demonstrated the ability to rapidly detect small metastatic LN preoperatively [80]. The median read time of USPIO-DW-MRI in each patient was about 13 min, significantly shorter than that of the traditional method [81]. Another strategy has been reported by the Liu group [82], where USPIO-PEG-sLeX molecular probes were constructed based on the conjugation between poly(ethylene glycol) (PEG) coated USPIO and Sialyl Lewis X (sLeX), the high-affinity ligand for the selectin family. The following in vivo models showed that USPIO-PEG-sLeX successfully targeted E-selectin associated with cancer metastasis and enabled early detection of metastasis in nasopharyngeal carcinoma. These findings would help provide promising platforms for the design of novel USPIO-based nanoparticles for LN mapping. For further clarification, studies should be conducted to determine nanoparticle imaging properties, diagnostic roles, and therapeutic values in human cancers.

## Other emerging magnetic nanoparticles

**Fig. 2:**
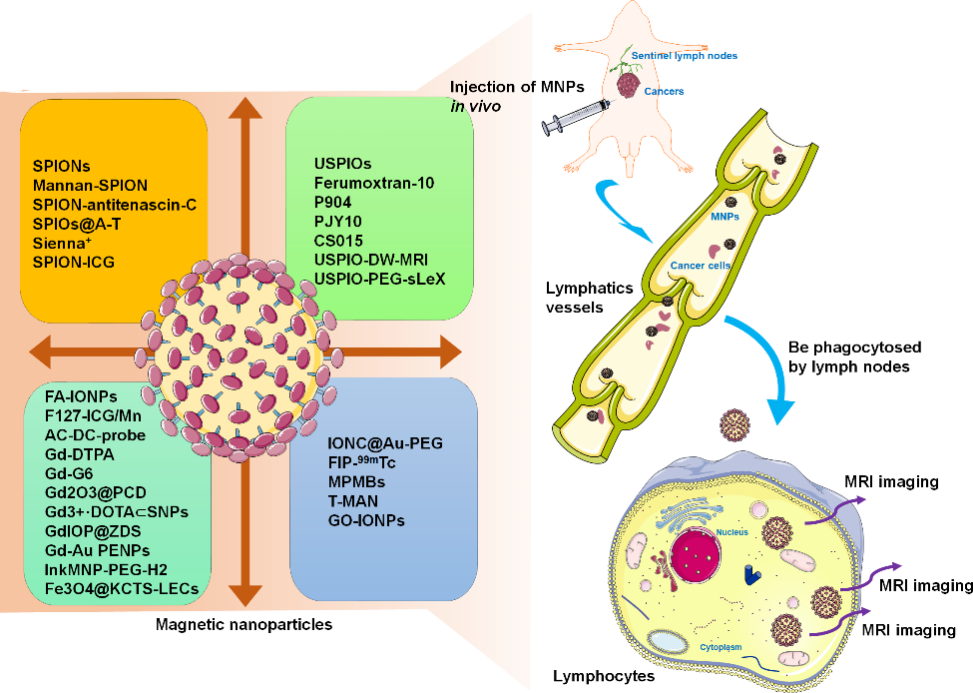
Schematic illustrations indicating the different types of MNPs in LN mapping. These MNPs with different physicochemical and pharmacodynamic characteristics have been successfully synthesized, and displayed the excellent diagnostic potential for identification of metastatic LNs in cancer patients

Newly designed MNPs with unique physicochemical and pharmacodynamic characteristics have been synthesized and demonstrated excellent diagnostic potential for lymph node mapping (Fig. 2; Table 3). For example, IONPs coated with folic acid (FA-IONPs) have been designed for LN imaging. Intracellular distribution analysis revealed the specific uptake of FA-IONPs by metastatic LNs that express the prostate-specific membrane antigen (PSMA). This folate transporter leads to the targeted detection of LNM by MRI in prostate cancer [83]. The combination of MagTrace and MagSeed, two non-radioactive magnetic techniques, showed the effectiveness and safety of the location of SLN in patients with breast cancer [84]. Recently, a clinical trial [NCT05569707, https://clinicaltrials.gov/] has been registered to evaluate the clinical application of handheld magnetic probes, Magtrace, in combination with SentiMag and differential magnetometry, in patients with melanoma. As alternative MRI agents, these emerging nanoparticles could be attractive for accurately identifying metastatic LN, providing a surgical roadmap for resectioning suspicious nodules [85]. Indeed, the visualization of LNs marked with nanoparticles would provide the foundation for future research to evaluate the underlying effects of MNPs for cancer research and treatment.

**Table 3:** Other emerging magnetic nanoparticles for imaging of metastatic lymph nodes

Nanoparticles	Injection	Models	Functions	Refs
FA-IONP	/	Cancer cells	Locating in the lymphatic system for the LNMs targeting	[83]
MagTrace and MagSeed	Subcutaneousl	Breast cancer patients	Displaying the effectiveness and safety for SLN localization	[84]
Fe3O4@KCTS-LECs-double antibody	Intravenous	Mouse model of colon cancer	Displaying fluorescence/MR dual-imaging for LECs and high tumor-targeted properties	[87]
F127-ICG/Mn	Subcutaneous	Mouse model of SLN metastasis	Successfully distinguishing the micro- and macro-SLN metastasis	[88]
AC-DC-probe	Endoscopic	Swine model of gastric cancer	ACDC-probe-labelled SLNs were clearly identified within AC and DC magnetic fields	[91]
Gd-DTPA	Subcutaneous	Mouse model of melanoma	Estimating the metastatic potential of tumor-draining LN cells	[93]
Gd-G6	Intracutaneous	Mouse model of breast cancer	Characterizing micro- and macro-metastasis within the lymphatic system	[95]
Gd_2_O_3_@PCD	Subcutaneous	Mouse model of breast cancer	Accurately in vivo staging of LNs and their metastatic spread	[97]
Gd^3+^·DOTA⊂SNPs	Intra-footpad	NOD/SCID mice		[98]
Gd-Au PENPs	Intravenous	VX2 tongue cancer model	Exhibiting the dual-modal CT/magnetic resonance lymphography properties	[100]
inkMNP-PEG-H2	Subcutaneous	4T1 tumor-bearing mice	High signal-to-noise ratio, high accumulation and high sensitivity in lymphatic imaging	[101]
MSN-probe	Intravenous	4T1 tumor metastatic model	Facilitating NIR optical, MRI and PET imaging simultaneously	[102]

**Fig. 3:**
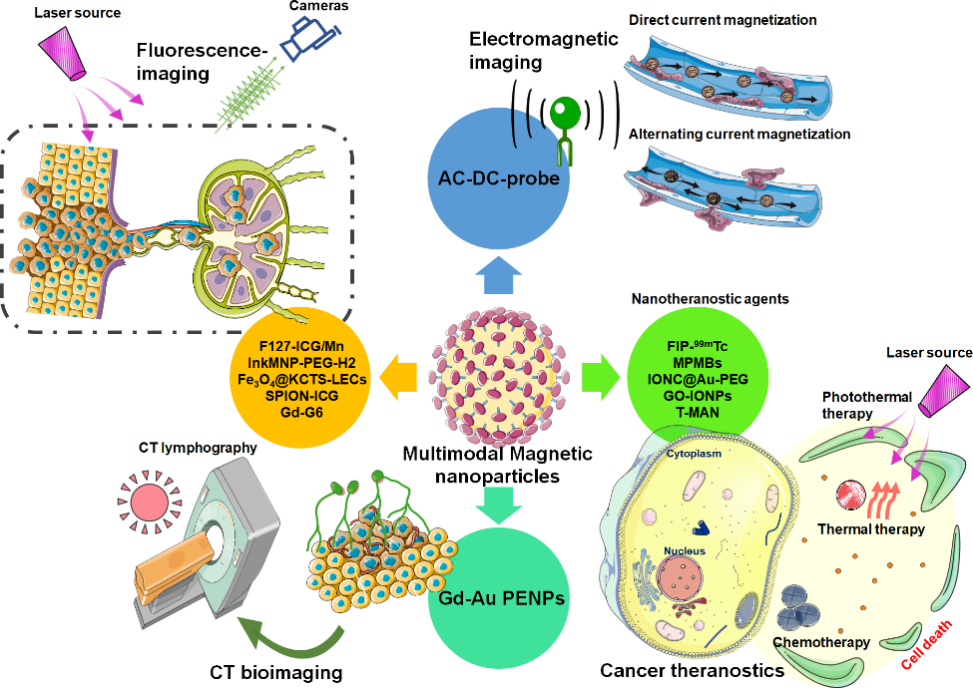
The magnetic nanoagents with multifunctional capability exhibit high sensitivity for nodal staging and cancer treatment. With the rapid development of technologies, several multimodal MNPs-based contrast imaging agents have been developed for the LNs detection and cancer theranostics

Due to the complement of different imaging modalities, nano agents with multimodal imaging capability exhibit high sensitivity and resolution for nodal staging **(**Fig. 3**)**. The use of dual-modal nanoparticles for fluorescent imaging and MRI of metastatic LNs is gradually attracting attention. Podoplanin and the lymphatic vessel endothelial hyaluronan receptor 1 (LYVE-1) are explicitly expressed in lymphatic endothelial cells (LECs), serving as particular lymphoid biomarkers [86]. Wu et al. have integrated podoplanin/LYVE-1 antibodies on the surface of Fe_3_O_4_-α-ketoglutarate chitosan nanocarriers to form the Fe_3_O_4_@KCTS-LEC double antibody nanoprobe [87]. Biochemical and functional assays suggested that these double antibody probes could select fluorescence/magnetic resonance dual imaging for LECs, allowing a precise diagnosis of lymphatic metastases. After tail vein injection, hematoxylin-eosin staining did not show prominent necrotic or inflammatory characteristics in the primary tissues of the mouse xenograft model of colon cancer, demonstrating its high biosafety. Fu et al. [88] prepared manganese porphyrin/indocyanine green (ICG) nanoparticles, F127-ICG/Mn, which efficiently integrated the F127-stabilized ICG and tetra(4-carboxyphenyl)porphyrin-Mn(III) (TCPP(Mn)). With the help of F127-ICG/Mn, the visualization of green-stained SLNs was evident, which could be seen with the naked eye. More importantly, micro- and macro-SLN metastasis could be successfully distinguished by fluorescence molecular imaging and magnetic particle imaging. During laparoscopic surgery, these dual-tracer SPION-ICG consisting of MNPs and ICG are more sensitive to detecting SLNs than MNPs or ICG alone [89]. Similarly, the dual-tracer strategy, ICG, and magnetic FerroTrace mixture could be used to identify reliable SLNs specifically and sensitively [90]. Furthermore, the clinical trial [NCT05092750] on FerroTrace’s SLN mapping has been registered in the ClinicalTrials.gov database (https://clinicaltrials.gov/), although no patients have been recruited. Kuwahata’s group [91] adopted a different approach to studying LN status. The combination of alternating current (AC) and direct current (DC) provides dual-tracer electromagnetic fields [92], thereby leading to more accurate imaging of SLNs. Consequently, Kuwahata et al. [91] developed a laparoscopic magnetic probe, an AC-DC probe. Gastric SLNs labeled with the AC-DC probe were detected within AC and DC magnetization. In laparoscopic surgery, SLN detection rates were not affected by the shine-through effect at the injection site.

In addition, Gadolinium (Gd)-based CAs (Gd-CAs) appear as one of the most advanced developments in the image-guided mapping of LNM during cancers. IONP conjugated with gadolinium diethylenetriamine pentaacetic acid (Gd-DTPA) was helpful in optimally estimating the metastatic potential of tumor-draining LN cells in the B16-F10 footpad melanoma model [93]. Administration of Gd-DTPA decreases the threshold size for nodule detection [94], indicating high sensitivity. Meanwhile, subcutaneous injection of generation-6 Gd dendrimer-based CAs (Gd-G6) could effectively characterize micro and macrometastasis within the lymphatic system [95]. After labeling with NIR Cy5.5fluorophore, Gd-G6 provided real-time intraoperative guidance for surgical SLN resection under MR and NIR imaging conditions [96]. Another two signal-enhanced and high-resolution Gd-based MRI agents, Gd_2_O_3_@PCD [97] and Gd^3+^·DOTA⊂SNPs [98] were successfully developed for the biological application of in vivo imaging. These injections of these two agents exhibited a crucial capacity for staging and diagnosing LNs and metastatic spread in tumor mice. With the rapid development of technologies, researchers have developed several multimodal Gd-based contrast imaging agents for detecting micro-metastatic LNs, such as GdIOP@ZDS nanoparticles [99]. Yang et al. [100] synthesized the Gd-loaded, polyethyleneimine-entrapped gold nano agents (Gd-Au PENPs) with attractive dual-modal CT/magnetic resonance lymphography properties. Functioning as an indirect CT/MRI CA, the Gd-Au PENPs possessed the ability to locate lingual SLN in rabbit VX2 tongue cancer models. Dong et al. [101] established a naked eye and NIR fluorescent nanoprobe inkMNP-PEG-H2 by connecting cuttlefish melanin with NIR fluorescent dye H2. When combined with Gd ions, inkMNP-PEG-H2 showed a high signal-to-noise ratio, accumulation, and sensitivity in lymphatic imaging. The embedding of the NIR dye ZW800 in nanoencapsulation labeled with Gd^3+^ and radionuclide ^64^Cu made it possible to facilitate optical, MRI, and PET NIR imaging simultaneously [102]. These triple-modal MSN probes have several excellent features, such as long intracellular retention time and high stability, contributing to long-term multimodal imaging for metastatic SLNs.

## Magnetic nanoparticle-guided imaging and therapeutic effects

Other projects were developed simultaneously for metastatic node imaging and cancer treatment (Fig. 3; Table 4). MNP-based molecular imaging probes provide excellent opportunities for early metastatic diagnosis and cancer treatment [103]. Gold-shelled magnetic IONPs were coated with PEG to obtain stable and superior IONC@Au-PEG in physiological solutions [104]. In the 4T1 tumor metastatic model, the IONC@Au-PEG nanocomposites could be transferred to SLNs via lymphatic pathways for imaging of cancer metastasis. Given that Au granules have an absorption peak in the NIR range, IONC@Au-PEG also functioned as a potential photothermal reagent possessing pronounced cytotoxic effects against primary tumors and SLNs. Through integrating magnetic Fe_3_O_4_, radioactive ^99m^Tc, and the NIR dye IR-1061, a research group from Soochow University successfully fabricated a new-style nanotheranostic agent, FIP-^99m^Tc [105]. The FIP-^99m^Tc nano agent, including MRI, PET/CT, and NIR fluorescence imaging, displayed excellent multimodal capability for LN mapping in breast cancer. Surprisingly, after laser irradiation, treatment with FIP-^99m^Tc could rapidly raise the temperature to exceed the threshold, facilitating the ablation of tumor cells within metastatic nodules. Multifunctional polymer microbubbles (MPMBs) could be artificially synthesized by encapsulating Fe_3_O_4_ loaded with chemotherapeutic doxorubicin in poly(lactic-co-glycolic acid) microbubbles [106]. Lymphographic MPMBs can quickly enter the lymphatic system and trigger the targeted delivery and controlled release of doxorubicin, which produces therapeutic effects on metastatic LNs. To facilitate the targeted delivery of nanoparticles to cancer cells, Shi’s group [107] reported a matrix metalloprotease-2 (MMP2) activatable Gd-doping-CuS micellar nanocluster (T-MAN). After injection, T-MAN accumulated preferentially in MMP2-positive cells of gastric tumors and metastatic sites, generating fluorescence/magnetic resonance bioimaging-guided photothermal therapy. Recently, magnetic compounds based on graphene nanomaterials, GO-IONPs, have been exploited to delineate RLNs in pancreatic cancer.

**Table 4:** Magnetic nanoparticles for the metastatic node imaging and cancer treatment

Nanoparticles	Injection	Models	Functions	Refs
IONC@Au-PEG	Intratumoral	4T1 tumor metastatic model	SLNs imaging; functioning as a potential photothermal reagent.	[104]
FIP-^99m^Tc	Intratumoral	4T1 tumor metastatic model	Multimodal imaging capability; facilitating the ablation of metastatic tumor cells	[105]
MPMBs	Intra-footpad	VX2 cancer model	Controlled release of doxorubicin into metastatic LNs	[106]
T-MAN	Intravenous	Orthotopic gastric tumor model	Targeted delivery; generating fluorescence/MRI-guided photothermal therapy	[107]
GO-IONPs	Intra-footpad	Mouse model of LN metastasis	Leading to clear delineation and photothermal ablation of RLNs	[108]

Meanwhile, GO-IONP-mediated photothermal therapy exhibited an excellent ability to photothermally ablate RLN and cancer cells [108]. Applications of these nano-theranostic agents could provide promising strategies for the imaging and treatment of primary tumors and their metastatic nodules in general. In particular, the design of multimodality imaging-based tumor biomarker-targeted nanocomposites holds great promise, as it can offer enhanced specificity for cancer theranostics in clinical management.

## Implications and future directions

As demonstrated by the reports highlighted in this review, the appearance of MNPs has already revolutionized our understanding of LN mapping. The development of MNPs, including IONPs, has proposed a promising functional platform that displays the capability to detect tumor metastasis early. As a helpful platform, these MNPs could also carry several different ligands for cancer imaging and treatment [109]. The incorporation of multiple components into magnetic nanomaterials allows the delivery of cargo to manage disease progression sensitively. In addition, the targeted transport of drugs or probes to disease sites can potentially weaken adverse effects [110]. The successful synthesis of a radio-labeled folate-mediated delivery system allowed for the integration of SPION and the chemotherapeutic drug docetaxel. This delivery system demonstrated remarkable distribution efficiency within folate receptor-positive tumor cells [111]. A Fe_3_O_4_-based magnetocaloric nanocarrier based on nanoparticles was designed to carry 4-amino-2-pentylselenoquinazoline and exhibited high specificity and selectivity against various cancer cells [112]. Kim et al. [113] constructed a thioketal-linked amphiphilic nano-assembly (MTS) to encapsulate hydrophobic manganese oxide (HMO). After intravenous injection, these MTS@HMO nanoparticles could be effectively absorbed by tumor cells, consequently inducing significant hydrogen peroxide scavenging and improving radiotherapy-mediated tumor elimination. Thus, we proposed that these magnetic systems could be developed to elucidate the potential activities of anti-cancer agents. However, some critical issues should be carefully addressed before magnetic nanomaterials are approved as theranostic compounds for clinical application.

As a rule, MNPs have been considered innocuous and biocompatible methods for biological applications in vivo. During the medication period, MNPs are relatively safe and well tolerated. Apart from some mild or moderate events, such as headache, back pain, vasodilatation, and urticaria, no serious adverse effects have been observed after injection of ferumoxtran-10 at the diagnostic dose (2.6 mg Fe/kg body weight) [56]. However, in rats injected with very high dose levels of ferumoxtran-10 (126–400 mg Fe/kg), Bourrinet et al. observed treatment-related toxic effects, such as breathing difficulties, swollen snout, darkening of the body areas, etc. [114]. These unexpected risks to chronic health might be due to iron-initiated oxidative stress, which ultimately damages cellular macromolecules and organelles [115]. In bronchial epithelial cells, treatment with Fe_3_O_4_ nanoparticles could generate lipid peroxidation and oxidative damage by promoting intracellular glutathione consumption and decreasing glutathione-S-transferase activity [116]. Under oxidative stress, iron-induced lipid peroxidation is closely related to the cellular production of reactive oxygen species (ROS) potentially harmful to living organisms [117].

Similarly, Gamal et al. found that MNPs could alter the activity of antioxidant enzymes and stimulate ROS production, ultimately leading to damage due to oxidative stress in the rat testes [118]. However, coating MNPs with chemical modification might alleviate their toxic impacts. Cellular experimental evidence revealed the minor effects of PEG-modified SPIONs on the biological behaviors of human fibroblasts [119]. Due to their prominent stability and negligible cytotoxicity, mannan-coated SPIONs have great potential for specific tracking of cancer cells in SLNs [29]. Polycaprolactone/chitosan-covered iron oxide prolonged the survival time of tumor-bearing mice without any toxic responses [120]. Therefore, these tests have indicated that the coating materials can effectively protect MNPs, enhancing their stability and demonstrating distinguished biocompatibility. Furthermore, the exploration and development of stabilized micelles open the possibility of IONPs as alternative MRI CAs for bioimaging [121].

Although MNP-based approaches have strong potential for the early diagnosis of MLN in cancer patients, their distribution in body fluids or cells has yet to be clarified. Surface properties have an important influence on the distribution, elimination, and bioavailability of MNPs [122]. Among these, the surface charge of MNPs is a critical element in controlling the final intracellular distribution [123]. The surface charge of the MNPs was mainly analyzed using the zeta potential [124]. Commercial MNPs frequently possess an intrinsic negative charge [125]. To explain this phenomenon, Al-Obaidy and colleagues [126] evaluated the physiochemical characteristics of SPIONs in vitro and in vivo. They found that the negative zeta-potential charge of SPIONs effectively prevented the formation of aggregates, resulting in suitable polydispersity ratios. IONPs with a surface charge of -31 mV exhibit good colloidal stability under physiological conditions [127]. However, compared to negatively charged MNPs, cultured cells more easily internalized the positively charged nanoparticles. Using quantitative methods, Calatayud et al. [128] demonstrated that almost 100% of positive MNPs were absorbed by SH-SY5Y human neuroblastoma cell lines, while the uptake rates of negative MNPs remained less than 50%. Such differences might be due to electrostatic interactions between the negative charge of the cell outer membrane and the positive charge of the nanoparticles [129]. Further investigations are required regarding the surface charge, which will become a pivotal issue in the biomedical fields of MNPs.

## Conclusions

The discovery of cancer cell dissemination in SLN has established lymphatic vessels as one of the frequent pathways for cancer cell metastasis. Furthermore, the state of the lymph nodes is recognized as a crucial prognostic and therapeutic factor for cancer patients. Many studies have emphasized the biological significance of LN diagnosis in carcinogenesis and have highlighted the positive impact of LN resection in treating cancer patients with LN infiltration. As a result, there is an urgent need to explore useful technical methods for the preoperative and intraoperative confirmation of macro and micro-metastatic LNs. The emergence of nanomedicine technologies has introduced novel and promising nanoprobes, such as MNPs, for molecular imaging of metastatic LNs.

## Data Availability

The data used to support this review are included in this article.

## Abbreviations

LNM: lymph node metastasis
LNs: lymph nodes
MRI: magnetic resonance imaging
CT: computed tomography
RLNs: regional lymph nodes
PET/CT: positron emission tomography/computed tomography
MNPs: magnetic nanoparticles
IONP: iron oxide nanoparticle
NIR: near infrared
SPIONs: superparamagnetic iron oxide nanoparticles
CAs: contrast agents
USPIOs: ultrasmall superparamagnetic iron oxides
PEG: poly(ethyleneglycol)
sLeX: Sialyl Lewis X
PSMA: prostate specific membrane antigen
LECs: lymphatic endothelial cells
ICG: indocyanine green
Gd: gadolinium
MMP2: matrix metalloprotease-2

## References

[1] Angelousi A, Hayes AR, Chatzellis E, Kaltsas GA, Grossman AB (2022). Metastatic medullary thyroid carcinoma: a new way forward. Endocrine-related Cancer.

[2] Jiang T, Xie L, Zhou S, Liu Y, Huang Y, Mei N (2022). Metformin and histone deacetylase inhibitor based anti-inflammatory nanoplatform for epithelial-mesenchymal transition suppression and metastatic tumor treatment. J Nanobiotechnol.

[3] Sadrkhanloo M, Entezari M, Orouei S, Ghollasi M, Fathi N, Rezaei S (2022). STAT3-EMT axis in tumors: modulation of cancer metastasis, stemness and therapy response. Pharmacol Res.

[4] Yang Y, Gu J, Li X, Xue C, Ba L, Gao Y (2021). HIF-1alpha promotes the migration and invasion of cancer-associated fibroblasts by miR-210. Aging and Disease.

[5] Zheng H, Yuan C, Cai J, Pu W, Wu P, Li C (2022). Early diagnosis of breast cancer lung metastasis by nanoprobe-based luminescence imaging of the pre-metastatic niche. J Nanobiotechnol.

[6] Qiao C, Wang H, Guan Q, Wei M, Li Z (2022). Ferroptosis-based nano delivery systems targeted therapy for colorectal cancer: insights and future perspectives. Asian J Pharm Sci.

[7] Chambers AF, Groom AC, MacDonald IC (2002). Dissemination and growth of cancer cells in metastatic sites. Nat Rev Cancer.

[8] Sugiyama S, Iwai T, Baba J, Oguri S, Izumi T, Sekino M (2021). MR lymphography with superparamagnetic iron oxide for sentinel lymph node mapping of N0 early oral cancer: a pilot study. Dento Maxillo Fac Radiol.

[9] Jayaprakasam VS, Alvarez J, Omer DM, Gollub MJ, Smith JJ, Petkovska I (2023). Watch-and-wait Approach to rectal Cancer: the role of imaging. Radiology.

[10] Davenport MS. Incidental findings and low-value care. AJR Am J Roentgenol. 2023:1–7. 10.2214/AJR.22.28926 36629303

[11] Bowen SR, Nyflot MJ, Gensheimer M, Hendrickson KR, Kinahan PE, Sandison GA (2012). Challenges and opportunities in patient-specific, motion-managed and PET/CT-guided radiation therapy of lung cancer: review and perspective. Clin Translational Med.

[12] Thoeny HC, Barbieri S, Froehlich JM, Turkbey B, Choyke PL (2017). Functional and targeted lymph node imaging in prostate Cancer: current Status and Future Challenges. Radiology.

[13] Pouw JJ, Grootendorst MR, Klaase JM, van Baarlen J, Ten Haken B (2016). Ex vivo sentinel lymph node mapping in colorectal cancer using a magnetic nanoparticle tracer to improve staging accuracy: a pilot study. Colorectal Disease: The Official Journal of the Association of Coloproctology of Great Britain and Ireland.

[14] Donohoe KJ, Carroll BJ, Chung DKV, Dibble EH, Diego E, Giammarile F (2023). Summary: Appropriate Use Criteria for Lymphoscintigraphy in Sentinel Node Mapping and Lymphedema/Lipedema. Journal of nuclear medicine: official publication. Soc Nuclear Med.

[15] Montagna G, Zhang J, Sevilimedu V, Charyn J, Abbate K, Gomez EA (2022). Risk factors and racial and ethnic disparities in patients with breast Cancer-related Lymphedema. JAMA Oncol.

[16] Gallo J, Long NJ, Aboagye EO (2013). Magnetic nanoparticles as contrast agents in the diagnosis and treatment of cancer. Chem Soc Rev.

[17] Han M, Kang R, Zhang C (2022). Lymph node mapping for Tumor Micrometastasis. ACS Biomaterials Science & Engineering.

[18] Kurochkin MA, German SV, Abalymov A, Vorontsov Dcapital AC, Gorin DA, Novoselova MV (2022). Sentinel lymph node detection by combining nonradioactive techniques with contrast agents: state of the art and prospects. J Biophotonics.

[19] Feldman AS, McDougal WS, Harisinghani MG (2008). The potential of nanoparticle-enhanced imaging. Urol Oncol.

[20] Lai G, Rockall AG. Lymph node imaging in gynecologic malignancy. Seminars in ultrasound, CT, and MR. 2010;31(5):363–76. 10.1053/j.sult.2010.07.006 20974356

[21] Fortuin AS, Bruggemann R, van der Linden J, Panfilov I, Israel B, Scheenen TWJ et al. Ultra-small superparamagnetic iron oxides for metastatic lymph node detection: back on the block. Wiley Interdisciplinary Reviews Nanomedicine and Nanobiotechnology. 2018;10(1). 10.1002/wnan.1471 PMC5763341 28382713

[22] Harisinghani M, Ross RW, Guimaraes AR, Weissleder R (2007). Utility of a new bolus-injectable nanoparticle for clinical cancer staging. Neoplasia.

[23] Cho HR, Choi SH, Lee N, Hyeon T, Kim H, Moon WK (2012). Macrophages homing to metastatic lymph nodes can be monitored with ultrasensitive ferromagnetic iron-oxide nanocubes and a 1.5T clinical MR scanner. PLoS ONE.

[24] Lei J, Xue HD, Li Z, Li S, Jin ZY (2010). Possible pathological basis for false diagnoses of lymph nodes by USPIO-enhanced MRI in rabbits. J Magn Reson Imaging: JMRI.

[25] Zhou Z, Chen H, Lipowska M, Wang L, Yu Q, Yang X (2013). A dual-modal magnetic nanoparticle probe for preoperative and intraoperative mapping of sentinel lymph nodes by magnetic resonance and near infrared fluorescence imaging. J Biomater Appl.

[26] Ittrich H, Peldschus K, Raabe N, Kaul M, Adam G (2013). Superparamagnetic iron oxide nanoparticles in biomedicine: applications and developments in diagnostics and therapy. RoFo: Fortschr auf dem Gebiete der Rontgenstrahlen und der Nuklearmedizin.

[27] Néel L (1949). Théorie du traînage magnétique des ferromagnétiques en grains fins avec application aux terres cuites. Ann de Géophysique.

[28] Vangijzegem T, Lecomte V, Ternad I, Van Leuven L, Muller RN, Stanicki D et al. Superparamagnetic Iron Oxide Nanoparticles (SPION): from Fundamentals to State-of-the-art innovative applications for Cancer Therapy. Pharmaceutics. 2023;15(1). 10.3390/pharmaceutics15010236 PMC9861355 36678868

[29] Vu-Quang H, Yoo MK, Jeong HJ, Lee HJ, Muthiah M, Rhee JH (2011). Targeted delivery of mannan-coated superparamagnetic iron oxide nanoparticles to antigen-presenting cells for magnetic resonance-based diagnosis of metastatic lymph nodes in vivo. Acta Biomater.

[30] Visscher M, Pouw JJ, van Baarlen J, Klaase JM, Ten Haken B (2013). Quantitative analysis of superparamagnetic contrast agent in sentinel lymph nodes using ex vivo vibrating sample magnetometry. IEEE Trans Bio Med Eng.

[31] Lai CH, Yen TC, Ng KK (2010). Surgical and radiologic staging of cervical cancer. Curr Opin Obst Gynecol.

[32] Lee Y, Lee JS, Kim CM, Jeong JY, Choi JI, Kim MJ (2008). Area of paradoxical signal drop after the administration of superparamagnetic iron oxide on the T2-weighted image of a patient with lymphangitic metastasis of the liver. Magn Reson Imaging.

[33] Kitamura N, Kosuda S, Araki K, Tomifuji M, Mizokami D, Shiotani A (2012). Comparison of animal studies between interstitial magnetic resonance lymphography and radiocolloid SPECT/CT lymphoscintigraphy in the head and neck region. Ann Nucl Med.

[34] Will O, Purkayastha S, Chan C, Athanasiou T, Darzi AW, Gedroyc W (2006). Diagnostic precision of nanoparticle-enhanced MRI for lymph-node metastases: a meta-analysis. Lancet Oncol.

[35] Grootendorst DJ, Jose J, Fratila RM, Visscher M, Velders AH, Ten Haken B (2013). Evaluation of superparamagnetic iron oxide nanoparticles (Endorem(R)) as a photoacoustic contrast agent for intra-operative nodal staging. Contrast Media Mol Imaging.

[36] Pantiora E, Tasoulis MK, Valachis A, Eriksson S, Kuhn T, Karakatsanis A (2023). Evolution and refinement of magnetically guided sentinel lymph node detection in breast cancer: meta-analysis. Br J Surg.

[37] Minamiya Y, Ito M, Hosono Y, Kawai H, Saito H, Katayose Y (2007). Subpleural injection of tracer improves detection of mediastinal sentinel lymph nodes in non-small cell lung cancer. Eur J cardio-thoracic Surgery: Official J Eur Association Cardio-thoracic Surg.

[38] Zhang F, Zhu L, Huang X, Niu G, Chen X (2013). Differentiation of reactive and tumor metastatic lymph nodes with diffusion-weighted and SPIO-enhanced MRI. Mol Imaging Biology.

[39] Grootendorst DJ, Fratila RM, Visscher M, Haken BT, van Wezel RJ, Rottenberg S (2013). Intra-operative ex vivo photoacoustic nodal staging in a rat model using a clinical superparamagnetic iron oxide nanoparticle dispersion. J Biophotonics.

[40] Johnson L, Pinder SE, Douek M (2013). Deposition of superparamagnetic iron-oxide nanoparticles in axillary sentinel lymph nodes following subcutaneous injection. Histopathology.

[41] Winter A, Kowald T, Engels S, Wawroschek F (2020). Magnetic resonance sentinel lymph node imaging and magnetometer-guided intraoperative detection in Penile Cancer, using Superparamagnetic Iron Oxide Nanoparticles: first results. Urol Int.

[42] Motoyama S, Ishiyama K, Maruyama K, Narita K, Minamiya Y, Ogawa J (2012). Estimating the need for neck lymphadenectomy in submucosal esophageal cancer using superparamagnetic iron oxide-enhanced magnetic resonance imaging: clinical validation study. World J Surg.

[43] Motomura K, Ishitobi M, Komoike Y, Koyama H, Noguchi A, Sumino H (2011). SPIO-enhanced magnetic resonance imaging for the detection of metastases in sentinel nodes localized by computed tomography lymphography in patients with breast cancer. Ann Surg Oncol.

[44] Motomura K, Izumi T, Tateishi S, Sumino H, Noguchi A, Horinouchi T (2013). Correlation between the area of high-signal intensity on SPIO-enhanced MR imaging and the pathologic size of sentinel node metastases in breast cancer patients with positive sentinel nodes. BMC Med Imaging.

[45] Gupta S, Rajesh A (2014). Magnetic resonance imaging of penile cancer. Magn Reson Imaging Clin N Am.

[46] Li X, Xu C, Yu Y, Guo Y, Sun H (2021). Prediction of lymphovascular space invasion using a combination of tenascin-C, cox-2, and PET/CT radiomics in patients with early-stage cervical squamous cell carcinoma. BMC Cancer.

[47] Song J, Hu Q, Huang J, Chen T, Ma Z, Shi H (2018). MR targeted imaging for the expression of tenascin-C in cervical cancer. Br J Radiol.

[48] Wang G, Li W, Shi G, Tian Y, Kong L, Ding N (2022). Sensitive and specific detection of breast cancer lymph node metastasis through dual-modality magnetic particle imaging and fluorescence molecular imaging: a preclinical evaluation. Eur J Nucl Med Mol Imaging.

[49] Cleaveland P, Lau M, Parnham A, Murby B, Ashworth D, Manohoran P (2019). Testing the feasibility of SentiMag/Sienna + for detecting Inguinal Sentinel Nodes in Penile Cancer (SentiPen): an eUROGEN and National Cancer Research Institute Trial. Eur Urol.

[50] Sreedhar S, Maloney J, Hudson S (2021). Introducing SentiMag in a rural setting: a 5-year experience. ANZ J Surg.

[51] Pouw JJ, Grootendorst MR, Bezooijen R, Klazen CA, De Bruin WI, Klaase JM (2015). Pre-operative sentinel lymph node localization in breast cancer with superparamagnetic iron oxide MRI: the SentiMAG Multicentre Trial imaging subprotocol. Br J Radiol.

[52] Nune SK, Gunda P, Majeti BK, Thallapally PK, Forrest ML (2011). Advances in lymphatic imaging and drug delivery. Adv Drug Deliv Rev.

[53] Ganeshalingam S, Koh DM (2009). Nodal staging. Cancer Imaging: The Official Publication of the International Cancer Imaging Society.

[54] Griffin LR, Frank C, Rao S, Seguin B. Lymphotropic nanoparticle magnetic resonance imaging for diagnosing metastatic lymph nodes in dogs with malignant head and neck tumours. Veterinary and comparative oncology. 2023. 10.1111/vco.12901 37186437

[55] Jain R, Dandekar P, Patravale V (2009). Diagnostic nanocarriers for sentinel lymph node imaging. J Controlled Release: Official J Controlled Release Soc.

[56] Anzai Y, Piccoli CW, Outwater EK, Stanford W, Bluemke DA, Nurenberg P (2003). Evaluation of neck and body metastases to nodes with ferumoxtran 10-enhanced MR imaging: phase III safety and efficacy study. Radiology.

[57] Shen N, Tan J, Wang P, Wang J, Shi Y, Lv W (2014). Indirect magnetic resonance imaging lymphography identifies lymph node metastasis in rabbit pyriform sinus VX2 carcinoma using ultra-small super-paramagnetic iron oxide. PLoS ONE.

[58] Triantafyllou M, Studer UE, Birkhauser FD, Fleischmann A, Bains LJ, Petralia G (2013). Ultrasmall superparamagnetic particles of iron oxide allow for the detection of metastases in normal sized pelvic lymph nodes of patients with bladder and/or prostate cancer. Eur J Cancer.

[59] Choi SH, Kim KH, Moon WK, Kim HC, Cha JH, Paik JH (2010). Comparison of lymph node metastases assessment with the use of USPIO-enhanced MR imaging at 1.5 T versus 3.0 T in a rabbit model. J Magn Reson Imaging: JMRI.

[60] Oghabian MA, Gharehaghaji N, Amirmohseni S, Khoei S, Guiti M (2010). Detection sensitivity of lymph nodes of various sizes using USPIO nanoparticles in magnetic resonance imaging. Nanomedicine: Nanatechnol Biology Med.

[61] Philips BWJ, Stijns RCH, Rietsch SHG, Brunheim S, Barentsz JO, Fortuin AS (2019). USPIO-enhanced MRI of pelvic lymph nodes at 7-T: preliminary experience. Eur Radiol.

[62] Froehlich JM, Triantafyllou M, Fleischmann A, Vermathen P, Thalmann GN, Thoeny HC (2012). Does quantification of USPIO uptake-related signal loss allow differentiation of benign and malignant normal-sized pelvic lymph nodes?. Contrast Media Mol Imaging.

[63] Fortuin AS, Smeenk RJ, Meijer HJ, Witjes AJ, Barentsz JO (2014). Lymphotropic nanoparticle-enhanced MRI in prostate cancer: value and therapeutic potential. Curr Urol Rep.

[64] Fortuin A, van Asten J, Veltien A, Philips B, Hambrock T, Johst S (2023). Small suspicious lymph nodes detected on Ultrahigh-field magnetic resonance imaging (MRI) in patients with prostate Cancer with high risk of nodal metastases: the First In-patient study on Ultrasmall Superparamagnetic Iron Oxide-enhanced 7T MRI. Eur Urol.

[65] Pultrum BB, van der Jagt EJ, van Westreenen HL, van Dullemen HM, Kappert P, Groen H (2009). Detection of lymph node metastases with ultrasmall superparamagnetic iron oxide (USPIO)-enhanced magnetic resonance imaging in oesophageal cancer: a feasibility study. Cancer Imaging: The Official Publication of the International Cancer Imaging Society.

[66] de Gouw D, Maas MC, Slagt C, Muhling J, Nakamoto A, Klarenbeek BR (2020). Controlled mechanical ventilation to detect regional lymph node metastases in esophageal cancer using USPIO-enhanced MRI; comparison of image quality. Magn Reson Imaging.

[67] Griffin L, Frank CB, Seguin B (2020). Pilot study to evaluate the efficacy of lymphotropic nanoparticle enhanced MRI for diagnosis of metastatic disease in canine head and neck tumours. Vet Comp Oncol.

[68] Kim SH, Oh SN, Choi HS, Lee HS, Jun J, Nam Y (2016). USPIO enhanced lymph node MRI using 3D multi-echo GRE in a rabbit model. Contrast Media Mol Imaging.

[69] Pandharipande PV, Mora JT, Uppot RN, Goehler A, Braschi M, Halpern EF (2009). Lymphotropic nanoparticle-enhanced MRI for independent prediction of lymph node malignancy: a logistic regression model. AJR Am J Roentgenol.

[70] Zamecnik P, Israel B, Feuerstein J, Nagarajah J, Gotthardt M, Barentsz JO (2022). Ferumoxtran-10-enhanced 3-T magnetic resonance angiography of pelvic arteries: initial experience. Eur Urol Focus.

[71] Fortuin AS, Deserno WM, Meijer HJ, Jager GJ, Takahashi S, Debats OA (2012). Value of PET/CT and MR lymphography in treatment of prostate cancer patients with lymph node metastases. Int J Radiat Oncol Biol Phys.

[72] Tatsumi Y, Tanigawa N, Nishimura H, Nomura E, Mabuchi H, Matsuki M (2006). Preoperative diagnosis of lymph node metastases in gastric cancer by magnetic resonance imaging with ferumoxtran-10. Gastric cancer: Official Journal of the International Gastric Cancer Association and the Japanese Gastric Cancer Association.

[73] Tokuhara T, Tanigawa N, Matsuki M, Nomura E, Mabuchi H, Lee SW (2008). Evaluation of lymph node metastases in gastric cancer using magnetic resonance imaging with ultrasmall superparamagnetic iron oxide (USPIO): diagnostic performance in post-contrast images using new diagnostic criteria. Gastric cancer: Official Journal of the International Gastric Cancer Association and the Japanese Gastric Cancer Association.

[74] Schilham MGM, Zamecnik P, Prive BM, Israel B, Rijpkema M, Scheenen T (2021). Head-to-Head comparison of (68)Ga-Prostate-specific membrane Antigen PET/CT and Ferumoxtran-10-Enhanced MRI for the diagnosis of Lymph Node Metastases in prostate Cancer patients. Journal of nuclear medicine: official publication. Soc Nuclear Med.

[75] Harisinghani MG, Dixon WT, Saksena MA, Brachtel E, Blezek DJ, Dhawale PJ (2004). MR lymphangiography: imaging strategies to optimize the imaging of lymph nodes with ferumoxtran-10. Radiographics: A Review Publication of the Radiological Society of North America Inc.

[76] Yoo RE, Cho HR, Choi SH, Won JK, Kim JH, Sohn CH (2014). Optimization of ultrasmall superparamagnetic iron oxide (P904)-enhanced magnetic resonance imaging of lymph nodes: initial experience in a mouse model. Anticancer Res.

[77] Yoo RE, Choi SH, Cho HR, Jeon BS, Kwon E, Kim EG (2014). Magnetic resonance imaging diagnosis of metastatic lymph nodes in a rabbit model: efficacy of PJY10, a new ultrasmall superparamagnetic iron oxide agent, with monodisperse iron oxide core and multiple-interaction ligands. PLoS ONE.

[78] Nie Y, Rui Y, Miao C, Li Q, Hu F, Gu H (2020). A stable USPIO capable for MR lymphography with ultra-low effective dosage. Nanomedicine: Nanatechnol Biology Med.

[79] Miao C, Hu F, Rui Y, Duan Y, Gu H (2019). A T(1)/T(2) dual functional iron oxide MRI contrast agent with super stability and low hypersensitivity benefited by ultrahigh carboxyl group density. J Mater Chem B.

[80] Birkhauser FD, Studer UE, Froehlich JM, Triantafyllou M, Bains LJ, Petralia G (2013). Combined ultrasmall superparamagnetic particles of iron oxide-enhanced and diffusion-weighted magnetic resonance imaging facilitates detection of metastases in normal-sized pelvic lymph nodes of patients with bladder and prostate cancer. Eur Urol.

[81] Thoeny HC, Triantafyllou M, Birkhaeuser FD, Froehlich JM, Tshering DW, Binser T (2009). Combined ultrasmall superparamagnetic particles of iron oxide-enhanced and diffusion-weighted magnetic resonance imaging reliably detect pelvic lymph node metastases in normal-sized nodes of bladder and prostate cancer patients. Eur Urol.

[82] Liu L, Liu L, Li Y, Huang X, Gu D, Wei B (2019). Ultrasmall superparamagnetic nanoparticles targeting E-selectin: synthesis and effects in mice in vitro and in vivo. Int J Nanomed.

[83] Bonvin D, Bastiaansen JAM, Stuber M, Hofmann H, Mionic Ebersold M (2017). Folic acid on iron oxide nanoparticles: platform with high potential for simultaneous targeting, MRI detection and hyperthermia treatment of lymph node metastases of prostate cancer. Dalton Trans.

[84] van Spiekerman MA, van Haaren ERM, Aldenhoven L, Frotscher CNA, Korver-Steeman R, van Bastelaar J (2023). An adapted protocol for magnetic localisation of nonpalpable breast cancer lesions and sentinel lymph nodes using a magnetic seed and superparamagnetic iron oxide tracer. J Surg Oncol.

[85] Ahmed M, Douek M (2014). What is the future of magnetic nanoparticles in the axillary management of breast cancer?. Breast Cancer Res Treat.

[86] Huuska N, Netti E, Lehti S, Kovanen PT, Niemela M, Tulamo R (2022). Lymphatic vessels are present in human saccular intracranial aneurysms. Acta Neuropathol Commun.

[87] Wu S, Liu X, He J, Wang H, Luo Y, Gong W (2019). A dual targeting magnetic nanoparticle for Human Cancer Detection. Nanoscale Res Lett.

[88] Fu X, Fu S, Cai Z, Jin R, Xia C, Lui S (2022). Manganese porphyrin/ICG nanoparticles as magnetic resonance/fluorescent dual-mode probes for imaging of sentinel lymph node metastasis. J Mater Chem B.

[89] Mihara K, Matsuda S, Nakamura Y, Aiura K, Kuwahata A, Chikaki S (2021). Intraoperative laparoscopic detection of sentinel lymph nodes with indocyanine green and superparamagnetic iron oxide in a swine gallbladder cancer model. PLoS ONE.

[90] Cousins A, Krishnan S, Krishnan G, Pham N, Milanova V, Nelson M (2023). Preclinical evaluation of sentinel node localization in the stomach via mannose-labelled magnetic nanoparticles and indocyanine green. Surg Endosc.

[91] Kuwahata A, Tanaka R, Matsuda S, Amada E, Irino T, Mayanagi S (2020). Development of magnetic probe for Sentinel Lymph Node detection in laparoscopic Navigation for gastric Cancer patients. Sci Rep.

[92] Belyaev VK, Rodionova VV, Grunin AA, Inoue M, Fedyanin AA (2020). Magnetic field sensor based on magnetoplasmonic crystal. Sci Rep.

[93] Partridge SC, Kurland BF, Liu CL, Ho RJ, Ruddell A (2015). Tumor-induced lymph node alterations detected by MRI lymphography using gadolinium nanoparticles. Sci Rep.

[94] Zhang F, Huang X, Qian C, Zhu L, Hida N, Niu G (2012). Synergistic enhancement of iron oxide nanoparticle and gadolinium for dual-contrast MRI. Biochem Biophys Res Commun.

[95] Kosaka N, Bernardo M, Mitsunaga M, Choyke PL, Kobayashi H (2012). MR and optical imaging of early micrometastases in lymph nodes: triple labeling with nano-sized agents yielding distinct signals. Contrast Media Mol Imaging.

[96] Koyama Y, Talanov VS, Bernardo M, Hama Y, Regino CA, Brechbiel MW (2007). A dendrimer-based nanosized contrast agent dual-labeled for magnetic resonance and optical fluorescence imaging to localize the sentinel lymph node in mice. J Magn Reson Imaging: JMRI.

[97] Rasouli Z, Riyahi-Alam N, Khoobi M, Haghgoo S, Gholibegloo E, Ebrahimpour A (2022). Lymph node metastases detection using gd(2)O(3)@PCD as Novel Multifunctional contrast Imaging Agent in Metabolic magnetic resonance Molecular Imaging. Contrast Media Mol Imaging.

[98] Chen KJ, Wolahan SM, Wang H, Hsu CH, Chang HW, Durazo A (2011). A small MRI contrast agent library of gadolinium(III)-encapsulated supramolecular nanoparticles for improved relaxivity and sensitivity. Biomaterials.

[99] Zhou Z, Liu H, Chi X, Chen J, Wang L, Sun C (2015). A Protein-Corona-Free T(1)-T(2) dual-modal contrast Agent for Accurate Imaging of Lymphatic Tumor Metastasis. ACS Appl Mater Interfaces.

[100] Yang Y, Zhou B, Zhou J, Shi X, Sha Y, Wu H (2018). Assessment of lingual sentinel lymph nodes metastases using dual-modal indirect CT/MR lymphography with gold-gadolinium-based nanoprobes in a tongue VX(2) carcinoma model. Acta Otolaryngol.

[101] Dong J, Sun J, Cai W, Guo C, Wang Q, Zhao X (2022). A natural cuttlefish melanin nanoprobe for preoperative and intraoperative mapping of lymph nodes. Nanomedicine: Nanatechnol Biology Med.

[102] Huang X, Zhang F, Lee S, Swierczewska M, Kiesewetter DO, Lang L (2012). Long-term multimodal imaging of tumor draining sentinel lymph nodes using mesoporous silica-based nanoprobes. Biomaterials.

[103] Qiao R, Liu C, Liu M, Hu H, Liu C, Hou Y (2015). Ultrasensitive in vivo detection of primary gastric tumor and lymphatic metastasis using upconversion nanoparticles. ACS Nano.

[104] Liang C, Song X, Chen Q, Liu T, Song G, Peng R (2015). Magnetic field-enhanced photothermal ablation of Tumor Sentinel Lymph Nodes to inhibit Cancer Metastasis. Small.

[105] Cai W, Fan G, Zhou H, Chen L, Ge J, Huang B (2020). Self-assembled hybrid nanocomposites for Multimodal Imaging-Guided Photothermal Therapy of Lymph Node Metastasis. ACS Appl Mater Interfaces.

[106] Niu C, Wang Z, Lu G, Krupka TM, Sun Y, You Y (2013). Doxorubicin loaded superparamagnetic PLGA-iron oxide multifunctional microbubbles for dual-mode US/MR imaging and therapy of metastasis in lymph nodes. Biomaterials.

[107] Shi H, Sun Y, Yan R, Liu S, Zhu L, Liu S (2019). Magnetic Semiconductor Gd-Doping CuS Nanoparticles as Activatable Nanoprobes for Bimodal Imaging and targeted photothermal therapy of gastric tumors. Nano Lett.

[108] Wang S, Zhang Q, Luo XF, Li J, He H, Yang F (2014). Magnetic graphene-based nanotheranostic agent for dual-modality mapping guided photothermal therapy in regional lymph nodal metastasis of pancreatic cancer. Biomaterials.

[109] Zhang C, Wang M, Zhang J, Zou B, Wang Y (2023). Self-template synthesis of mesoporous and biodegradable Fe(3)O(4) nanospheres as multifunctional nanoplatform for cancer therapy. Colloids and Surfaces B Biointerfaces.

[110] Pusta A, Tertis M, Craciunescu I, Turcu R, Mirel S, Cristea C. Recent advances in the development of drug delivery applications of magnetic nanomaterials. Pharmaceutics. 2023;15(7). 10.3390/pharmaceutics15071872 PMC10383769 37514058

[111] Cetin O, Gungor B, Ichedef C, Parlak Y, Bilgin ES, Ustun F (2023). Development of a radiolabeled folate-mediated drug delivery system for effective delivery of Docetaxel. ACS Omega.

[112] Galarreta-Rodriguez I, Etxebeste-Mitxeltorena M, Moreno E, Plano D, Sanmartin C, Megahed S et al. Preparation of Selenium-Based Drug-Modified Polymeric Ligand-Functionalised Fe(3)O(4) Nanoparticles as Multimodal Drug Carrier and Magnetic Hyperthermia Inductor. Pharmaceuticals. 2023;16(7). 10.3390/ph16070949 PMC10385492 37513861

[113] Kim S, Sundaram A, Mathew AP, Hareshkumar VS, Mohapatra A, Thomas RG et al. In situ hypoxia modulating nano-catalase for amplifying DNA damage in radiation resistive colon tumors. Biomaterials Sci. 2023. 10.1039/d3bm00618b 37504889

[114] Bourrinet P, Bengele HH, Bonnemain B, Dencausse A, Idee JM, Jacobs PM (2006). Preclinical safety and pharmacokinetic profile of ferumoxtran-10, an ultrasmall superparamagnetic iron oxide magnetic resonance contrast agent. Invest Radiol.

[115] Yarjanli Z, Ghaedi K, Esmaeili A, Rahgozar S, Zarrabi A (2017). Iron oxide nanoparticles may damage to the neural tissue through iron accumulation, oxidative stress, and protein aggregation. BMC Neurosci.

[116] Zhang W, Gao J, Lu L, Bold T, Li X, Wang S (2021). Intracellular GSH/GST antioxidants system change as an earlier biomarker for toxicity evaluation of iron oxide nanoparticles. NanoImpact.

[117] Kicheeva AG, Sushko ES, Bondarenko LS, Kydralieva KA, Pankratov DA, Tropskaya NS et al. Functionalized Magnetite Nanoparticles: characterization, Bioeffects, and role of reactive oxygen species in Unicellular and Enzymatic Systems. Int J Mol Sci. 2023;24(2). 10.3390/ijms24021133 PMC9861541 36674650

[118] Gamal A, Kortam LE, El Ghareeb AEW, El Rahman HAA (2022). Assessment of the potential toxic effect of magnetite nanoparticles on the male reproductive system based on immunological and molecular studies. Andrologia.

[119] Gupta AK, Curtis AS (2004). Surface modified superparamagnetic nanoparticles for drug delivery: interaction studies with human fibroblasts in culture. J Mater Sci - Mater Med.

[120] Kiamohammadi L, Asadi L, Shirvalilou S, Khoei S, Khoee S, Soleymani M (2021). Physical and Biological Properties of 5-Fluorouracil Polymer-Coated Magnetite Nanographene Oxide as a new thermosensitizer for alternative magnetic hyperthermia and a magnetic resonance imaging contrast Agent: in Vitro and in vivo study. ACS Omega.

[121] Andrade RGD, Veloso SRS, Castanheira EMS. Shape Anisotropic Iron Oxide-Based magnetic nanoparticles: synthesis and Biomedical Applications. Int J Mol Sci. 2020;21(7). 10.3390/ijms21072455 PMC7178053 32244817

[122] Gupta AK, Wells S (2004). Surface-modified superparamagnetic nanoparticles for drug delivery: preparation, characterization, and cytotoxicity studies. IEEE Trans Nanobiosci.

[123] Amigoni L, Salvioni L, Sciandrone B, Giustra M, Pacini C, Tortora P et al. Impact of tuning the surface charge distribution on Colloidal Iron Oxide Nanoparticle toxicity investigated in Caenorhabditis elegans. Nanomaterials. 2021;11(6). 10.3390/nano11061551 PMC8230852 34208275

[124] Hajiali S, Daneshjou S, Daneshjoo S (2022). Biomimetic synthesis of iron oxide nanoparticles from Bacillus megaterium to be used in hyperthermia therapy. AMB Express.

[125] Szymczyk A, Drozd M, Kaminska A, Matczuk M, Trzaskowski M, Mazurkiewicz-Pawlicka M et al. Comparative evaluation of different Surface Coatings of Fe(3)O(4)-Based magnetic Nano Sorbent for Applications in the nucleic acids extraction. Int J Mol Sci. 2022;23(16). 10.3390/ijms23168860 PMC9408759 36012139

[126] Al-Obaidy R, Haider AJ, Al-Musawi S, Arsad N (2023). Targeted delivery of paclitaxel drug using polymer-coated magnetic nanoparticles for fibrosarcoma therapy: in vitro and in vivo studies. Sci Rep.

[127] Dutta B, Rawoot YA, Checker S, Shelar SB, Barick KC, Kumar S (2020). Micellar assisted aqueous stabilization of iron oxide nanoparticles for curcumin encapsulation and hyperthermia application. Nano-Structures & Nano-Objects.

[128] Calatayud MP, Sanz B, Raffa V, Riggio C, Ibarra MR, Goya GF (2014). The effect of surface charge of functionalized Fe3O4 nanoparticles on protein adsorption and cell uptake. Biomaterials.

[129] Imran M, Ehrhardt CJ, Bertino MF, Shah MR, Yadavalli VK. Chitosan stabilized silver nanoparticles for the Electrochemical detection of Lipopolysaccharide: a facile Biosensing Approach for Gram-Negative Bacteria. Micromachines. 2020;11(4). 10.3390/mi11040413 PMC7231338 32295278

